# Comparison of one‐step with two‐step production of *Bacillus atrophaeus* spores for use as bioindicators

**DOI:** 10.1002/mbo3.1332

**Published:** 2022-11-03

**Authors:** Philipp Stier, Ulrich Kulozik

**Affiliations:** ^1^ Chair of Food and Bioprocess Engineering, TUM School of Life Sciences Technical University of Munich Freising Germany

**Keywords:** *Bacillus*, bioindicator, spore production, sterilization validation

## Abstract

The production method of spores significantly influences the resistance of spores used as bioindicators (BI) in the validation of sterilization of packaging material surfaces in aseptic food manufacturing. Therefore, the standardization of the spore production method represents an important and desirable goal in industrial BI production to ensure reliable validation test results. Previously, we recommended a two‐step production approach for submerged spore production, in which the cultivation phase to obtain high cell mass was separate from the sporulation phase. In this work, a one‐step manufacturing process was investigated to reduce production complexity and facilitate standardization of spore production. It was found that one‐step BI production is technically possible but at the expense of spore yield. The two‐step manufacturing process can realize almost 10‐fold higher spore yields.

## Graphical abstract

The production method of spores significantly influences the resistance of spores used as bioindicators (BI) in the validation of sterilization of packaging material surfaces in aseptic food manufacturing. Therefore, the standardization of the spore production method represents an important and desirable goal in industrial BI production to ensure reliable validation test results. A one‐step manufacturing process was investigated in this work to reduce production complexity. Compared to the recommended two‐step manufacturing process, almost 10‐fold higher yields can be realized.

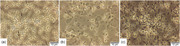

## 1 INTRODUCTION

In the food and pharmaceutical industries, bioindicators (BI) consisting of spores of *Bacillus atrophaeus* or *Geobacillus stearothermophilus* are used to assess the inactivation performance of sterilization processes, for example, of packaging material treatment with liquid or vaporized hydrogen peroxide (FDA, 2007; McLeod et al., 2017; VDMA, 2021) before aseptic filling. It is known that the sporulation conditions can cause a significant change in the spore structure and, thus, in the resistance of the spores (Abhyankar et al., 2016; Bressuire‐Isoard et al., 2016, 2018; Isticato et al., 2020; Setlow, 2014). This means that the manufacturing method significantly influences the resistance of the BI, which may result in differences in the inactivation result and, thus, in uncertainties in the validity of sterilization process test results when using BI with variable and, most times, unknown resistances against the applied sterilization method. Therefore, standardization of BI production would be essential and a great step forward to ensure consistent quality and resistance of spores. Currently, state‐of‐the‐art is that there are no agreed standardized BI production protocols, including the selection of nutrient contents, cell growth, and sporulation conditions.

We already showed that spore resistance can be influenced and predicted in submerged production or solid‐state production on agar plates as a function of sporulation temperature and sporulation pH (Stier & Kulozik, 2020; Stier et al., 2021). Compared to the solid‐state method, which typically results in a high degree of cell differentiation due to inconsistent conditions across and through the agar plate (Vlamakis et al., 2008), submerged production offers more consistent fermentation conditions. In our experiments on the submerged production of *B. atrophaeus* spores in the bioreactor (Stier & Kulozik, 2020), we observed during fermentation that sporulation occurred almost simultaneously for all vegetative cells and thus under uniform sporulation conditions. In addition, sporulation is faster (2 days in submerged bioreactor production vs. approximately 10 days on agar plates), and spore harvesting and purification from fermentation broth residues were reported to be easier and more consistent (Eschlbeck et al., 2017; Eschlbeck & Kulozik, 2017; Stier & Kulozik, 2020; Stier et al., 2021).

Furthermore, more parameters influencing spore resistance can be controlled in the bioreactor than in the solid‐state process on agar plates, for instance, pH and oxygen conditions, which can vary considerably in solid‐state production. The BI manufacturing industry has recognized these advantages, and companies have partially switched production to the submerged process (anonymous, oral source from our industry partners). However, standardization of BI production conditions has still not been achieved; therefore, the issue of different BI resistances remains. In our previous work (Stier & Kulozik, 2020), submerged spore production was investigated in a two‐step procedure, where the cultivation phase of vegetative cells and the sporulation phase to form spores were separated. As a cultivation medium for *B. atrophaeus*, *Terrific Broth* (TB) and a modified version of *Difco Sporulation Medium* (DSM) as sporulation medium were used. As the results demonstrated, this has the advantage of generating vital vegetative cells with the desired high cell numbers before exposing them to the sporulation medium, which in its composition, was optimized to induce sporulation rapidly and simultaneously for all cells. This differs from other works that reported a one‐step approach combining cultivation and sporulation. For example, in the production of *B. subtilis* spores, Nguyen Thi Minh et al. (2011) used the sporulation medium already to culture the vegetative cells. Upon finalization of limited cell growth, the authors continued sporulation in the same medium and had to over inoculate the volume of the preculture for spore production into the same sporulation medium to achieve the desired high number of spores. Since there was no washing step between cultivation and sporulation in the same medium required, this procedure can be described as a one‐step method, although technically, there were steps transferring the cells at the end of cultivation into another bioreactor for sporulation. Eschlbeck & Kulozik (2017) did not over inoculate but combined the phase of cultivation and sporulation in a single production step simply by continuing the cultivation step to achieve sporulation in the same bioreactor using the same medium. This, however, resulted in a low spore yield.

Avoiding two separate production steps (i.e., the elimination of cultivation and sporulation in separate media) represents a substantial simplification of spore production. This way, it could have corresponding advantages in industrial BI production and facilitate standardization. The purpose of this work, therefore, was to test the one‐step in a head‐to‐head comparison versus the two‐step method.

To address the potential for optimization of spore production, we investigate the possibility of the one‐step production process for its application in BI production. We combined this approach with testing the suitability of different established sporulation media types for different *Bacillus* species, modified, however, in their glucose contents to allow for more cell growth, on cultivating vegetative cells before the onset of sporulation and compared these results with cell growth in several cultivation media. The reason for increasing the glucose concentration in classical sporulation media with typically low glucose contents to induce starvation was that the vegetative cells should not sporulate immediately after cell growth when inoculated into the bioreactor to adapt to the sporulation conditions.

Finally, we reviewed the sporulation yield of *B. atrophaeus* in a one‐step process in different sporulation media and compared the results with those obtained in our previous work (Stier & Kulozik, 2020), applying the two‐step method with separated cultivation and sporulation steps.

## 2 EXPERIMENTAL PROCEDURES

### 2.1 Strain

Spores of the strain *B. atrophaeus* ATCC 9372, which has been established as a hydrogen peroxide BI (FDA, 2007; McLeod et al., 2017; VDMA, 2021), was used as starting material. The spores were of the same production batch and stored at aliquots of 500 µl with a concentration of 10^7^ colony‐forming units (CFU)/ml at −80°C to ensure the initial characteristics of the spores did not change. The aliquots enabled the spores to be taken without thawing the rest of the spore suspension.

### 2.2 Media

The following media were used for cultivation or, depending on the experiment, for the sporulation of *B. atrophaeus*. These media are typical growth media used in microbiology (Lysogeny Broth‐Lennox [LB], Casein Peptone Soymeal Peptone Medium [CASO], Super Broth [SB], and TB) and established sporulation media for *Bacillus* spp. (Bacillus Sporulation Medium [BSM], DSM, and Minimal Sporulation Medium [MSM]). Most of the single ingredients used for formulating the media were from Merck KGaA, unless mentioned otherwise.

*LB* comprised of casein peptone 10 g/L (for microbiology, Gerbu Biotechnik GmbH), yeast extract 5 g/L (for microbiology), and NaCl 5 g/L (ACS reagent, ≥99.0%).

*CASO* was made of casein peptone 17 g/L (for microbiology, Gerbu Biotechnik GmbH), K_2_HPO_4_ 2.5 g/L (ACS reagent, ≥99%), glucose 2.5 g/L (for biochemistry, Reag. Ph. Eur., 97.5%–102.0%), NaCl 5 g/L (ACS reagent, ≥99.0%), and soy peptone 3 g/L (for microbiology, Gerbu Biotechnik GmbH).

*SB* consisted of casein peptone 35 g/L (for microbiology, Gerbu Biotechnik GmbH), yeast extract 20 g/L (for microbiology), and NaCl 5 g/L (ACS reagent, ≥99.0%).

*TB* contained casein peptone 12 g/L (for microbiology, Gerbu Biotechnik GmbH), yeast extract 24 g/L (for microbiology), K_2_HPO_4_ 9.4 g/L (ACS reagent, ≥98%), KH_2_PO_4_ 2.2 g/L (ACS reagent, ≥99%), and glycerol 8 g/L (Rotipuran ≥ 99.5%, Carl Roth GmbH & Co. KG).

*BSM* (modified in glucose content, based on Posada‐Uribe et al. [2015]) comprised of glucose 3 g/L (for biochemistry, Reag. Ph. Eur., 97.5%–102.0%), MgSO_4_·7H_2_O 0.59 g/L (ACS ≥99%, Carl Roth GmbH & Co. KG), KH_2_PO_4_ 6 g/L (ACS reagent, ≥98%, Merck KGaA), beef extract 5 g/L (for cell biology, Gerbu Biotechnik GmbH), casein peptone 3 g/L (for microbiology, Gerbu Biotechnik GmbH), NaCl 0.01 g/L (ACS reagent, ≥99.0%), and stock salt solution (0.1 M FeSO_4_·7H_2_O 1.136 ml/L (ACS reagent, ≥99%), 0.1 M ZnSO_4_·7H_2_O 300 µl/L (puriss. p.a., ACS reagent, reag. ISO, Reag. Ph. Eur., ≥99.5%), 0.1 M CaCl_2_ 9.9 ml/L (anhydrous, Supelco), and 0.1 M MnCl_2_∙4H_2_O 30 mL/L (ACS reagent, ≥98%). For BSM, the glucose content was adjusted to 3 g/L, analogous to the subsequent DSM. This ensures better comparability of the media with regard to the growth of *B. atrophaeus*, regardless of the glucose content present.

*DSM* (modified in glucose content, based on Harwood [1990]) comprised of casein peptone 5 g/L (for microbiology, Gerbu Biotechnik GmbH), beef extract 3 g/L (for cell biology, Gerbu Biotechnik GmbH), KCl 3.5 g/L (ACS reagent, 99.0%–100.5%), MgSO_4_·7H_2_O 0.25 g/L (ACS ≥99%, Carl Roth GmbH & Co. KG), 30% glucose 10 ml/L (for biochemistry, Reag. Ph. Eur., 97.5%–102.0%), 1 M Ca(NO_3_)_2_·4H_2_O 1 ml/L (ACS reagent, 99%), 10 mM MnCl_2_·4H_2_O 1 ml/L (ACS reagent, ≥98%) and 1 mM FeSO_4_·7H_2_O 1 ml/L (ACS reagent, ≥99%). The glucose content was increased to 3 g/L during media optimization based on our previous work (Stier & Kulozik, 2020), which was retained in this work for comparability.

*MSM* (modified for submerged use, based on Pruß et al. [2012]) comprised of casein peptone 5 g/L (for microbiology, Gerbu Biotechnik GmbH), beef extract 3 g/L (for cell biology, Gerbu Biotechnik GmbH) and MnSO_4_·H_2_O 10 mg/L (≥99%, p.a., ACS, Carl Roth GmbH & Co. KG). This medium was not initially designed for the submerged growth of *Bacillus* spp. and was, therefore, not included in the submerged cultivation experiments in the beginning. However, the medium was applied in the sporulation experiments because, as described in more detail in the discussion, BSM was unsuitable for sporulation. Thus another sporulation medium had to be used for comparison. To adapt the medium for submerged sporulation, agar‐agar was not added in the media preparation.

### 2.3 Cultivation of vegetative cells of *B. atrophaeus*

The aim was to compare the results from our previous work (Stier & Kulozik, 2020) applying two‐step spore production (cultivation in TB and sporulation in DSM) with the one‐step procedure according to Eschlbeck & Kulozik (2017) (cultivation and sporulation in one step in DSM). For this purpose, cells of *B. atrophaeus* were cultivated in both TB and DSM. Since other media could be more suitable for *B. atrophaeus* cultivation and to examine a broader spectrum of results regarding the suitability of media, the media SB, LB, CASO, and BSM were also included in the comparison.

To use the same starting material in all cultures, 100 ml of each medium (pH 7.2) was inoculated with 500 µl each of *B. atrophaeus* spores from the same production batch in a 250 ml baffled shake flask (Schott Duran® baffled culture flask, Erlenmeyer shape, straight neck for metal caps; Duran Group GmbH) as a biological triplicate. Incubation was performed at 30°C and 120 rpm for 22 h.

From 8 to 22 h of incubation, the optical density at 600 nm (OD_600_) was determined several times per hour. This time window was chosen because the most significant differences were expected here due to the progressive growth of the cells. In addition, the pH was checked every 4 h to exclude a negative influence of pH on cell growth since the pH was not controlled actively during the incubation.

### 2.4 Sporulation of *B. atrophaeus*

To examine the sporulation success in the one‐step manufacturing process, cells of *B. atrophaeus* were incubated in the sporulation media DSM and BSM until sporulation occurred. This means that no preliminary cultivation in a cultivation medium took place. Since it could already be concluded from the first part of the experiment that BSM appears unsuitable for the sporulation of *B. atrophaeus*, the additional medium MSM was used for a more comprehensive comparison.

Each 500 µl of *B. atrophaeus* spores from the same production batch were inoculated into 400 ml of the respective sporulation medium (pH 7.2) in a 1 L baffled shake flask (Schott Duran® baffled culture flask, Erlenmeyer shape, straight neck for metal caps; Duran Group GmbH) as a biological duplicate and incubated at 30°C and 120 rpm for 96 h. The cultivation volume was increased in this experiment compared to the experiments on the cultivation of vegetative cells still to identify enough spores in case of low spore yields. After 96 h of incubation, the total suspension volume was centrifuged (4000 *g*, 4°C, 10 min), and the supernatant was discarded. The pellet resulting from centrifugation was resuspended with Milli‐Q water.

To determine the spore concentration, a portion of this washed suspension was diluted with Ringer's solution (Ringer tablets for the production of sterile Ringer's solution, ¼ Ringer's solution, item no. MC1155250001, Merck KGaA) in duplicate and plated out on Plate Count Agar (casein peptone 0.5%, for microbiology, Gerbu Biotechnik GmbH; yeast extract 0.25%, for microbiology; glucose 0.1%, for biochemistry, Reag. Ph Eur., 97.5%–102.0%; agar‐agar 1.5%, for microbiology, Carl Roth GmbH & Co. KG; pH 7.0). After incubation for 48 h at 30°C, the number of CFU was determined. Because it was important for the rest of the experiment not to separate the free spores from the cell debris and the forespores and because a proportion of the free spores is always lost during purification (Stier & Kulozik, 2020; Stier et al., 2021), the spore concentration was determined with the non‐purified suspension immediately after the end of incubation to minimize the risk of regermination. The presence of living cells of the suspensions, which would falsify the spore concentration determination, was tested by phase‐contrast microscopy. Vital cells were observed as motile unicellular or bicellular cells (Kearns & Losick, 2005) in the cultivation phase, whereas the remaining cells floated motionless in the suspension after the sporulation. Additionally, this observation is supported by the fact that the cells were cultured for 96 h in a sporulation medium that no longer offered adequate living conditions at a certain point in time, which is why the cells sporulated. Therefore, the presence of living cells is highly unlikely even without phase microscopic evidence.

For phase‐contrast microscopic assessment of sporulation, the suspensions were concentrated by a factor of 10 to ease visual inspection since the spore density in the original suspension was too low for a meaningful comparison. The concentration was done by another centrifugation step (4000 *g*, 4°C, 10 min), discarding the supernatant and suspending the pellet with 1/10 Milli‐Q water of the original volume. The pellet resulting from centrifugation was comprised of three phases, with only the lowest phase at the bottom containing free spores, followed by forespores and cell debris (according to Stier & Kulozik, 2020; Stier et al., 2021). Therefore, to study sporulation success, it was necessary to keep all three phases intact when discarding the supernatant not to distort the result, respectively the ratio of spores to nonsporulated cells by discarding one of the upper phases and not to lose spores, which is to some extent unavoidable with this type of purification.

## 3 RESULTS AND DISCUSSION

**Figure 1:**
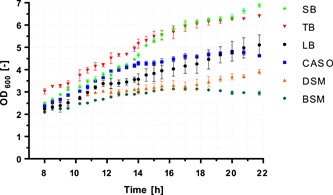
Optical densities (OD_600_) of cultures of *Bacillus atrophaeus* in the media SB, TB, LB, CASO, DSM, and BSM. BSM, Bacillus Sporulation Medium; CASO, Casein Peptone Soymeal Peptone Medium; DSM, Difco Sporulation Medium; LB, Lysogeny Broth‐Lennox; SB, Super Broth; TB, Terrific Broth.

First, the question was clarified whether the one‐step process could lead to similar results as the two‐step production. Since each vegetative cell can form only one spore, the cell density in the culture could already indicate the final maximally obtainable number of spores, provided that the sporulation medium allows the highest possible sporulation rate. For this reason, *B. atrophaeus* ATCC 9372 was cultivated in triplicate at 30°C, 120 rpm for 22 h in four cultivation media (SB, TB, LB, and CASO) and two sporulation media (DSM and BSM). The results are shown in Figure 1. The OD_600_ was determined at intervals between 8 and 22 h of cultivation. Growth progression from 0 to 8 h was not recorded because no major differences were expected in this time window.

After 8 h of incubation, slight differences in the cell densities of the cultures were already apparent. As expected, these differences increased during incubation. TB, as used in our last publication (Stier & Kulozik, 2020) as a cultivation medium before inoculation into the sporulation medium, showed the highest cell density after 8 h with an OD_600_ of ~3. The other cultures were in the range of OD_600_ ~2–2.5. As cell growth progressed, the differences in cell densities became increasingly pronounced. After approximately 14 h of incubation, the cell densities of the cultures in TB and SB converged and continued to show strong cell growth. In contrast, the cell density in CASO started to level off after 14 h. In LB, the cultures showed the lowest cell density among the pure cultivation media in the first 19 h of cultivation but finally caught up with CASO by the end of the incubation. After 22 h, cell densities of OD_600_ ~6.5 in TB, ~6.9 in SB, ~5.1 in LB, and ~4.5 (i.e., maximum of ~4.6 after 21 h) in CASO were finally reached in the cultivation media.

The sporulation media BSM and DSM produced the lowest cell densities over the entire growth course. In addition, the cell density in BSM decreased after approximately 17 h of incubation, while the cell density in DSM continued to increase. After 22 h of incubation, a cell density of OD_600_ ~3.9 was reached in DSM and ~2.9 in BSM (with a maximum OD_600_ of ~3.1 after 17 h). This shows that the sporulation media have limited suitability for cell cultivation since large cell densities could not be achieved. Based on the decrease in cell density in BSM, it can also be assumed that sporulation had already started.

To assess the state of sporulation in the sporulation media, the cell suspensions were checked by phase‐contrast microscopy at the end of the 22 h incubation. Surprisingly, no spores were found in either of the sporulation media. Therefore, the decrease in cell density in BSM cannot be explained by the formation of spores but probably by the beginning lysis of cells, triggered either by starvation or other milieu conditions. This is notable because BSM and DSM have relatively similar nutrient compositions. Despite their rather similar composition of the nutrient components, the cell density in DSM, in contrast to BSM, continued to increase in the course of growth. This could either be because casein peptone is preferred to beef extract, which is why the composition of DSM would be slightly better, or that the milieu conditions, that is, concentration and presence of ions in BSM compared to DSM, are of adverse effect for *B. atrophaeus*. The decrease in cell density in BSM could also mean that BSM is not well suited for the sporulation of *B. atrophaeus* ATCC 9372. Otherwise, the formation of spores would have to be expected in parallel with the decrease in cell density. However, the cells in this medium seem to have died before sporulation.

The data showed that cultivation is possible in all the media listed, although cell yields are significantly higher in the media dedicated to cell growth. This may be partly due to the significantly higher nutrient contents in the cultivation media compared to the sporulation media but also due to lower levels of specific ions. The pH value of the media (Table 1) is not expected to be a reason for growth stagnation since the changes were moderate within the incubation time.

**Table 1:** pH in the course of the incubation period in the media SB, TB, CASO, LB, DSM, and BSM

	pH (−)					
Incubation (h)	SB	TB	CASO	LB	DSM	BSM
10	7.03	6.75	7.20	7.41	7.44	7.34
14	6.90	6.67	7.26	7.58	7.69	7.51
18	6.71	6.61	7.41	7.83	8.05	7.63
21	6.50	6.60	7.60	7.99	8.23	7.90

*Note*: The initial pH was set to 7.2 in all media before inoculation.

Abbreviations: BSM, Bacillus Sporulation Medium; CASO, Casein Peptone Soymeal Peptone Medium; DSM, Difco Sporulation Medium; LB, Lysogeny Broth‐Lennox; SB, Super Broth; TB, Terrific Broth.

The next step was to examine whether the sporulation media were also suitable for the one‐step production process or could lead to similar results as the two‐step production process. Since cell growth in our past study (Stier & Kulozik, 2020) also took place in a shake flask and the vegetative cells were only transferred to a bioreactor for sporulation, this experiment was performed exclusively in a shake flask. This provided better comparability between the experiments because vegetative cell growth to sporulation in the bioreactor would have had a significantly different effect on the cultures.

**Figure 2:**
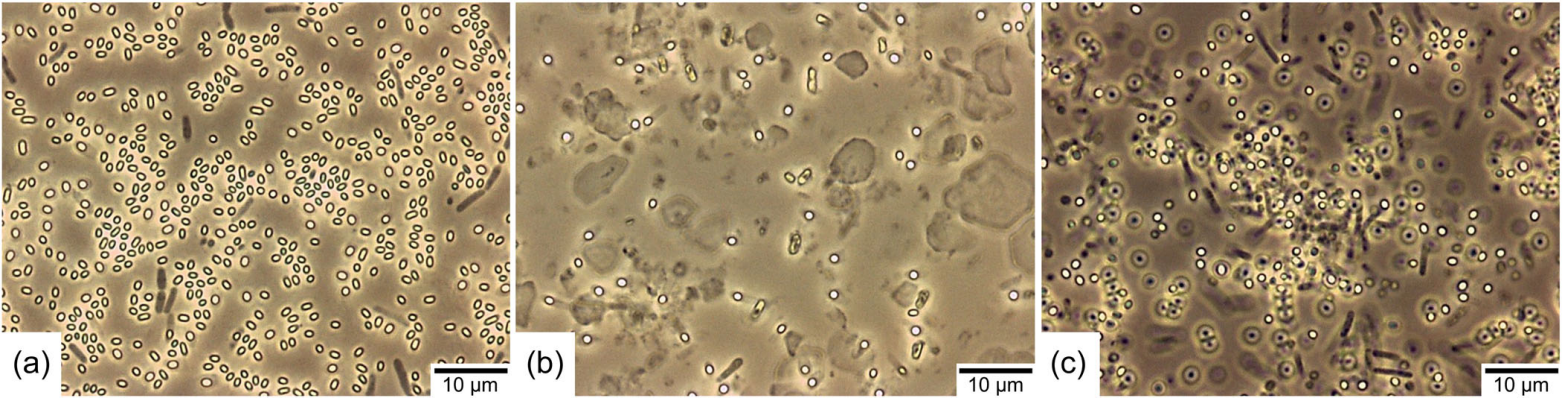
Phase‐contrast microscopy (×100) of the sporulation results of *Bacillus* *atrophaeus* after cultivation and sporulation after 96 h in the media (a) Difco Sporulation Medium (DSM), (b) Bacillus Sporulation Medium (BSM), and (c) Minimal Sporulation Medium (MSM). Spores can be observed as bright spots and dead cells as gray rods.

As described above, the cell density in BSM decreased at a certain time of incubation without spores forming. Due to this, we suspected that this medium could not be suitable for producing *B. atrophaeus* ATCC 9372 spores. We, therefore, extended the comparison and included MSM as another sporulation medium. In the previous cultivation media experiments, this medium was initially not included because it is a medium for obtaining spores using the solid‐state method on agar plates (Pruß et al., 2012). To allow submerged cultivation with this medium, the recipe was adjusted by omitting agar‐agar. The media were inoculated with 500 µl of *B. atrophaeus* spores from the same production batch and incubated for 96 h. Within this period, cultivation and sporulation occurred in the same medium without additional intervention. Figure 2 shows representative results of the double determination of spore formation in the respective sporulation media after 96 h of incubation.

As suspected, the spore yield in BSM (Figure 2b) was comparatively low. Only a few free spores could be found in this medium. In addition, a few dead cells (gray rods) were sighted, suggesting that the majority of the cells lysed, as suspected already in the first experiment. Significantly more spores were formed in MSM (Figure 2c). Although this medium was not intended for submerged spore production in its original recipe, it appears suitable for *B. atrophaeus* ATCC 9372. Nevertheless, not all cells sporulated in this medium, and a large proportion died. The medium DSM (Figure 2a) produced the best sporulation efficiency in this comparison. In this medium, the majority of cells appeared to be sporulated, and only a few dead cells were found. The absence of vegetative cells was tested by microscopy of several duplicate samples.

The spore suspensions of these sporulation media were also plated on Plate Count Agar to determine the spore concentration. Since there were no longer any vegetative cells in the suspensions, the results provided information about the pure number of germinable spores in the respective medium (1 CFU was set equivalent to 1 germinable spore). The spores were not heat‐activated before plating. In DSM, an average spore concentration of 1.6 × 10^8^ CFU/ml could be observed, in BSM 4.8 × 10^7^ CFU/ml, and in MSM 8.4 × 10^7^ CFU/ml (these spore concentrations correspond to the original spore suspension before concentration by a factor of 10 for phase‐contrast microscopic evaluation). These results confirm the results of the phase‐contrast microscopic evaluation that the highest number of spores was formed in DSM. Since the initial cell concentration applied for inoculation was identical in all media, this result shows that DSM produces the best sporulation efficiency among the tested sporulation media.

The results also show that the one‐step production process is, in principle, suitable for obtaining spores, depending on the sporulation medium selected. It is notable, though, that an OD_600_ ~3.9 was achieved in DSM during cultivation, and no decrease in growth or beginning sporulation was observed up to this point. In our previous procedure to produce *B. atrophaeus* spores (Stier & Kulozik, 2020), we inoculated the bioreactor with an OD_600_ of 1.0. Sporulation, therefore, started about 3 h later at a comparatively low cell density. Nevertheless, we achieved spore yields of 1.1 × 10^9^ CFU/ml in this two‐step production approach under similar sporulation conditions (30°C, pH 7.2, 30% pO_2_). Accordingly, overinoculation of vegetative cells from a cultivation medium into the sporulation medium seems to result in a more significant proportion of cells sporulating than in the one‐step manufacturing process. In our opinion, this is the only way to explain why, despite a lower cell density in the sporulation medium, we obtained a spore yield increased by a factor of almost 10 in the two‐step production process. This difference can be attributed to the best conditions during the cell growth phase in the two‐step method achieving high cell numbers at first before entering the sporulation phase, which was also performed under optimal conditions yielding high spore numbers and avoiding losses due to cell death. These results underline our previous recommendations for submerged production of *B. atrophaeus* spores for use as BI. Although the one‐step manufacturing procedure could reduce effort and cost, this is accompanied by significantly lower spore yields.

## 4 CONCLUSIONS

Standardization of BI production is essential to ensure consistent quality and resistance of spores. We have previously recommended a two‐step manufacturing process for producing spores for use as BI. However, a one‐step manufacturing process would have potential advantages in terms of effort and cost, which would potentially also enable standardization to be uniformly established by and between BI manufacturers. However, since the one‐step manufacturing process could obtain only low spore yields, we restate our previous recommendation to prefer the two‐step manufacturing process with separated cultivation and sporulation phases. Practical experience in aseptic food and pharmaceutical production shows that standardization of BI manufacturing is essential to reduce uncertainties of sterilization validation tests. An aspect to be addressed in future works is to test the resistance variability between the one‐step and the two‐step BI production methods. It has already been shown in our previous work (Stier & Kulozik, 2020) that resistance variability can be reduced by a better understanding of the relationship between sporulation conditions and spore resistance. Since suitable media and methods are now available as a result of this work, this will be tested in future experiments.

## AUTHOR CONTRIBUTIONS

**Philipp Stier**: Conceptualization (lead); data curation (lead); investigation (lead); methodology (lead); project administration (equal); validation (lead); visualization (lead); writing – original draft (lead); writing – review and editing (equal). **Ulrich Kulozik**: Conceptualization (supporting); funding acquisition (lead); project administration (equal); writing – review and editing (equal).

## CONFLICT OF INTEREST

None declared.

## ETHICS STATEMENT

None required.

## Data Availability

All data are provided in full in the results section of this paper, apart from the two‐step production data, which are available at https://doi.org/10.3390/molecules25132985.
